# Idebenone Protects against Spontaneous Chronic Murine Colitis by Alleviating Endoplasmic Reticulum Stress and Inflammatory Response

**DOI:** 10.3390/biomedicines8100384

**Published:** 2020-09-28

**Authors:** Sonia Shastri, Tanvi Shinde, Agampodi Promoda Perera, Nuri Gueven, Rajaraman Eri

**Affiliations:** 1 Gut Health Laboratory, School of Health Sciences, College of Health and Medicine, University of Tasmania, Launceston 7250, Tasmania, Australia; Tanvi.Shinde@utas.edu.au (T.S.); agampodi.perera@utas.edu.au (A.P.P.); 2 Centre for Food Innovation, Tasmanian Institute of Agriculture, University of Tasmania, Launceston 7250, Tasmania, Australia; 3 School of Pharmacy and Pharmacology, College of Health and Medicine, University of Tasmania, Hobart 7005, Tasmania, Australia; nuri.guven@utas.edu.au

**Keywords:** chronic colitis, endoplasmic reticulum stress, idebenone, mucin, pro-inflammatory cytokines, *Winnie*

## Abstract

Endoplasmic reticulum (ER) stress in intestinal secretory goblet cells has been linked to the development of ulcerative colitis (UC). Emerging evidence suggests that the short chain quinone drug idebenone displays anti-inflammatory activity in addition to its potent antioxidant and mitochondrial electron donor properties. This study evaluated the impact of idebenone in *Winnie* mice, that are characterized by spontaneous chronic intestinal inflammation and ER stress caused by a missense mutation in the mucin *MUC2* gene. Idebenone (200 mg/kg) was orally administered daily to 5–6 weeks old *Winnie* mice over a period of 21 days. Idebenone treatment substantially improved body weight gain, disease activity index (DAI), colon length and histopathology score. Immunohistochemistry revealed increased expression of MUC2 protein in goblet cells, consistent with increased *MUC2* mRNA levels. Furthermore, idebenone significantly reduced the expression of the ER stress markers C/EBP homologous protein (*CHOP*), activating transcription factor 6 (*ATF6*) and X-box binding protein-1 (*XBP-1*) at both mRNA and protein levels. Idebenone also effectively reduced pro-inflammatory cytokine levels in colonic explants. Taken together, these results indicate that idebenone could represent a potential therapeutic approach against human UC by its strong anti-inflammatory activity and its ability to reduce markers of ER stress.

## 1. Introduction

Ulcerative colitis (UC) and Crohn’s disease (CD) are the two major forms of inflammatory bowel disease (IBD) which are characterized by chronic and relapsing inflammation of the gastrointestinal tract, with an unclear etiology [1] that involves the interaction of environmental and genetic factors [2]. Body weight loss, bloody stool, abdominal cramps and diarrhea are the common symptoms often presented in IBD patients [3]. Additionally, IBD patients are also at risk of developing extraintestinal diseases and other inflammatory diseases such as colorectal cancer, asthma, bronchitis and multiple sclerosis [4,5]. Currently, IBD treatment primarily focuses on immunosuppressive therapies involving biologicals, corticosteroids and immunomodulators [6,7,8]. However, the side effects of these treatments limit their therapeutic potential [8,9,10,11]. Hence, there is an urgent need for pragmatic treatment options targeting the multifactorial recurrent inflammatory cascade to mitigate chronic intestinal inflammation.

Emerging evidence points to the potential presence of a pathophysiological circuit consisting of microbial dysbiosis, dysregulated immune response, altered barrier integrity, oxidative stress, genetic mutations and aberrant endoplasmic reticulum (ER) stress that all contribute to the development of intestinal inflammation [12,13,14,15,16,17]. A number of studies have illustrated the involvement of defective epithelial secretory goblet cells in the pathogenesis of IBD [18,19,20]. Mucin MUC2 is the major macromolecule synthesized by goblet cells that protects the intestine from harmful luminal toxins and microbes by producing a thick mucus layer over the mucosal surface [21]. The folding and N-glycosylation of MUC2 take place in the ER [22]. In UC, a decreased secretion of mucus due to a reduced number of functional goblet cells leads to the accumulation of unfolded MUC2 precursor protein in the secretory goblet cell. This accumulation is responsible for aberrant ER stress via the activation of the unfolded protein response (UPR) [18,23,24]. ER stress has been linked to the pathogenesis of human IBD [25]. Under physiological conditions, ER chaperon glucose regulated protein 78 (GRP78) is sequestered in ER with three transmembrane UPR proteins: activating transcription factor 6 (ATF6), protein kinase RNA-like endoplasmic reticulum kinase (PERK) and inositol-requiring enzyme 1 (IRE-1α and IRE-1β). Under stress conditions, GRP78 dissociates from these transmembranous proteins and binds to misfolded proteins in the ER [26,27,28,29,30]. This dissociation activates ATF6, PERK and IRE-1 and its related downstream signaling, which regulates the release of ER-stress associated transcription factors from the nucleus such as C/EBP homologous protein (CHOP) and X-box binding protein 1 (XBP-1), leading to the development of colitis [30,31,32,33,34]. Therefore, restoring intestinal homeostasis by ameliorating ER stress could be developed as a therapeutic strategy for IBD.

Several murine models connect ER stress with intestinal inflammation [18,23,35,36]. *Winnie*, a mouse model of spontaneous chronic inflammation, recapitulates the symptoms associated with human UC and includes a prominent innate/adaptive/Th17 immune response [37]. The chronic inflammation in *Winnie* is caused by a single point or missense mutation in the mucin *MUC2* gene which leads to mucin misfolding in the ER, resulting in UPR activation-linked ER stress. As a consequence, there is less production of MUC2 from goblet cells, a reduction in goblet cell size and numbers and a diminished mucus barrier, similar to human UC [18]. The inflammation in *Winnie* mice starts at the age of 6 weeks and progresses to severe colitis by the age of 16 weeks [38]. Like in human patients, colitis in *Winnie* is characterized by relapses and remissions [39]. The colonic damage in distal colon (DC) of *Winnie* is more prominent as compared to that in the proximal colon (PC) due to higher epithelial stress and more microbial dysbiosis [18,24,40]. Histopathology of the DC of *Winnie* mice revealed a UC-like phenotype that includes crypt abscesses, reduced goblet cell numbers, crypt elongations, mucosal surface erosion, infiltration of neutrophils and a distorted crypt architecture [18]. Recently, the *Winnie* model was successfully used to test several clinical drugs such as MCC950, glucocorticoids and thiopurines, and dietary fibers [24,40,41,42], that showed therapeutic effects comparable to UC patients.

Idebenone, a short chain benzoquinone, is described as a mitochondrial electron donor and a potent antioxidant [43]. In the 1980s, idebenone was developed for the treatment of dementia. Currently, it is marketed for the treatment of an inherited mitochondrial disorder in Europe [44] and is developed for neuromuscular indications. Idebenone is well tolerated and safe for human use as either acute or chronic treatment [45]. Previously, in a dextran sodium sulfate (DSS)-induced mouse model of acute colitis [46], idebenone ameliorated intestinal inflammation by an indirect antioxidant effect via upregulating NAD(P)H dehydrogenase quinone 1/superoxide dismutase (NQO-1/SOD) detoxifying enzymes, which was associated with reduced lipid peroxidation. In the same study, idebenone also preserved the expression of the barrier integrity proteins occludin and zona-occludin 1. In this colitis model, idebenone also reduced the levels of several pro-inflammatory cytokines and chemokines down to levels found in healthy animals [46]. This activity was also reported in a rat model of titanium-dioxide-induced kidney toxicity, where idebenone reduced the levels of interleukins 1, 6 (IL-1, IL-6) and tumor necrosis factor alpha (TNF-α), which was associated with improved kidney function [47]. Idebenone also reduced lipopolysaccharide (LPS) stimulated neuroinflammation in BV2 microglial cells by suppressing pro-inflammatory cytokines and inducible nitric oxide synthase (iNOS) [48]. In addition to its anti-inflammatory properties, idebenone reportedly downregulated the expression of the ER marker *CHOP* in the HtrA2 knock-out mice, which was associated with protection against neurodegeneration [49].

Based on this evidence and on our previous results [46], we hypothesized that idebenone would also confer protection against chronic colitis in the *Winnie* mouse model of spontaneous colitis. The current study highlights the protective role of idebenone in UC to ameliorate chronic inflammation by simultaneously suppressing ER stress and pro-inflammatory cytokine levels.

## 2. Materials and Methods

### 2.1. Animals

The *C57BL/6J* and *Winnie* mice of both sexes were obtained from the animal breeding facility of the University of Tasmania. All the animals were housed in a controlled temperature environment with a twelve-hour day and night cycle. All the animals underwent an acclimatization period of 7 days before performing any experiment. All the mice were caged individually with proper access to autoclaved drinking water ad libitum and normal chow pellets. All the experiments were conducted in accordance with the ethics approval from Animal Ethics Committee of University of Tasmania (approval number: A00016166 approved on 6 March 2017). All the procedures were conducted in accordance to the Australian Code of Practise for Care and Use of Animals for Scientific Purposes (8th Edition, 2013).

### 2.2. Study Experimental Design and Drug Treatment

Equal number of *C57BL/6J* mice (aged 5–6 weeks) and *Winnie* mice (aged 5–6 weeks) were divided into three groups: (1) *C57BL/6J* Healthy Controls without drug (HC), (2) *Winnie* Controls (Winnie) and (3) idebenone-treated *Winnie* (Winnie + I), with eight animals/group. For drug preparation, idebenone was suspended in 0.5% of carboxymethylcellulose (CMC), 4% sucrose and autoclaved powered chow pellets as mentioned previously [46]. The wet food mash containing idebenone was aliquoted as 2.5 g/dish and stored in −20 °C. *Winnie* controls received the drug vehicle in food mash, and idebenone containing food mash was administered to the *Winnie* plus idebenone-treated group for 21 days daily. Autoclaved normal drinking water was provided to all groups. All the mice were sacrificed on day 21.

### 2.3. Histopathological Analysis and Clinical Scoring

Disease Activity Index (DAI) was calculated daily by adding the individual scores of body weight, blood in stool and stool consistency over the whole period of the experiment, as mentioned previously [40,41,50]. Briefly, the following parameters were considered: body weight loss scores (0 = 0–1%, 1 = 1–5%, 2 = 6–10% and 3 = 11–15%), stool consistency scores (0 = hard/formed stool, 1 = soft/loose stool, 2 = very soft and 3 = watery stool/diarrhea) and bloody stool scores (0 = negative hemoccult/ no traces of blood, 1 = positive hemoccult, 2 = visible traces of blood and 3 = gross/rectal bleeding). The mice were dissected, and colons were cut longitudinally into two halves from which one half was utilized for histopathological analysis using the swiss roll technique, while the other half was snap-frozen for molecular analysis. All the swiss rolls were fixed in 10% *v*/*v* neutral-buffered formalin for 24 h, and subsequently transferred to 70% ethanol. They were embedded in paraffin wax and 5-µm thick tissue sections were cut using a rotary microtome. Paraffin-embedded tissue slides were then stained with hematoxylin and eosin (H&E) dyes and were used for histopathological grading in a blinded manner, as described previously [40,41,51]. Images were captured using a Leica microscope (DM500, Leica Microsystems, Mannheim, Germany).

### 2.4. Immunohistochemistry

Immunohistochemical analysis was performed to detect the localization of mucin MUC2 proteins using an HRP/DAB Detection IHC kit (ab64621, Abcam, Victoria, Australia), as mentioned previously [46,50]. Briefly, tissue section slides were dewaxed in xylene and rehydrated through a series of graded ethanol before incubating in citrate buffer (pH 6) for antigen retrieval process in a decloaking chamber at 121 °C for 4 min. The endogenous peroxidase activity was blocked by incubating the tissue slides with hydrogen peroxide block for 10 min, following protein blocking for a further 30 min at room temperature. Primary antibodies against MUC2 (1:1000, NBP1-31231, Novus Biologicals, Victoria, Australia) were used to incubate colonic tissue slides overnight at 4 °C. After washing the slides in phosphate buffer saline (1XPBS), slides were first incubated with biotinylated goat antirabbit IgG for 10 min, followed by incubation with Streptavidin-peroxidase conjugate for a further 10 min at room temperature. Slides were further incubated using DAB chromogen and substrate for 10 min according to manufacturer’s protocol. Slides were counterstained with hematoxylin and bluing in ammonia water before dehydrating in graded ethanol and clearing in xylene. Finally, the slides were mounted using DPX media and images were taken using a Leica DM500 microscope. Image Pro-Plus 7 software was used to examine the staining intensity by randomly choosing four different areas from one slide where *n* = 3 slides/group, in a blinded manner.

### 2.5. RNA Extraction and Gene Expression Analysis

The RNA from colonic tissue was extracted and purified using RNeasy Mini kit (Qiagen, Melbourne, Australia) according to the kit manual. The concentration of the extracted RNA was calculated using an Eppendorf Biophotometer. The extracted RNA was reverse transcribed to complementary cDNA using iScript cDNA synthesis kit (Biorad, New South Wales, Australia) according to the reaction conditions, as mentioned by manufacturer. Then, 100 nanogram of cDNA from each sample was added to a PCR reaction mixture containing Fast Advanced Master Mix (Applied Biosystems, Victoria, Australia) and a single gene-specific TaqMan probes or primers set (ThermoFisher Scientific, Victoria, Australia). The primers used were *GRP78* (Assay ID: Mm00517691_m1), *CHOP* (Assay ID: Mm00492097_m1), *ATF6* (Assay ID: Mm01295319_m1), *XBP-1* (Assay ID: Mm00457357_m1) and *PERK* (Assay ID: Mm00438700_m1), *MUC2* (Mm01276696_m1) and glyceraldehyde 3-phosphate dehydrogenase (*GAPDH)* (Assay ID: Mm99999915_g1).

Thermal cycling was performed using a StepOnePlus RT-qPCR instrument (Applied Biosystems, Victoria, Australia). Quantification of gene expression was done using a comparative method (ΔΔCT). The threshold cycle (CT) for each gene was normalized to GAPDH (house-keeping gene) and expressed as relative to the mean of related control group.

### 2.6. Lipid Peroxidation

The amount of malondialdehyde (MDA) levels present in colonic tissue was determined using lipid peroxidation colorimetric/fluorometric assay kit (K739, Bio Vision, New South Wales, Australia) as described previously [46,52]. The colon tissues were homogenized in the lysis buffer provided in the kit, followed by centrifugation at 13,000× *g* to collect supernatant. Next, thiobarbituric acid (TBA) was added to the resulting supernatant, which formed MDA-TBA adduct after boiling at 95 °C for 1 h. The absorbance of colored MDA-TBA adduct was detected colorimetrically at a wavelength of 532 nm in a spectrophotometer. The amount of MDA in the tissue samples was quantified by plotting a graph against the MDA standard calibration curve. The data were expressed as nmol/mg protein.

### 2.7. Western Blotting Analysis

For western blot analysis, frozen distal colon tissues were washed with PBS and lysed in radioimmunoprecipitation assay buffer (RIPA) containing protease inhibitor cocktails (Complete ULTRA Tablets, Mini, EDTA-free, New South Wales, Australia). The concentrations of protein in supernatants were determined according to the protocol described by the DC protein Assay Kit from Biorad. Tissue-extracted proteins lysates (20 µg) were suspended in loading buffer and separated on SDS-PAGE polyacrylamide gels (4–20%, Mini-Protean TGX Precast Gels, Bio-Rad, New South Wales, Australia) in a 1x electrophoresis buffer run at 100 V for 1 h. Subsequently, proteins from the gel were electro-transferred onto a polyvinylidene difluoride membrane (PVDF) using a wet-transfer system. The membranes were then blocked in 5% skim milk prepared in TBST and incubated overnight with primary antibody against NQO-1 (1:1000, ab34173, Abcam), GRP78 (1:1000, NBP1-06274, Novus biologicals) CHOP (1:1000, NBP2-13172, Novus Biologicals) and β-actin (1:8000, NB600-503, Novus Biologicals) at 4 °C. The antibodies were detected by incubating with Horseradish peroxidase (HRP)-conjugated secondary antibody (1:3000, 7074, Cell Signaling Technology, Queensland, Australia) at room temperature for 1 h. Protein bands were visualized using SuperSignal, West Pico PLUS chemiluminescent substrate (Thermo Scientific, Victoria, Australia). The images were captured, and the band densities relative to housekeeping gene (β-actin) were measured using LAS-3000 image reader version 2.2 (Fujifilm Luminescent Image Analyzer, Fuji Life Sciences, Tokyo, Japan).

### 2.8. Cytokine Measurement 

Explants from proximal and distal colon tissues from each group were excised, washed with PBS and cultured in RPMI 1640 culture medium containing 1% antibiotics solution (10 mg/mL streptomycin and 10,000 U/mL penicillin, Sigma-Aldrich Pty. Ltd., New South Wales, Australia) and 10% *v*/*v* fetal bovine serum (Gibco Life Technologies Pty. Ltd., Victoria, Australia) in a 12-well plate, as described previously [40,41,50,53]. After overnight incubation, supernatant was collected, centrifuged and stored at −80 °C until further analysis. Cytokine concentrations in the colonic explants were determined using Bio-plex Pro Mouse cytokine 23-plex kit (catalogue number: #M60009RDPD, Bio-Rad, New South Wales, Australia), according to manufacturer’s protocol, using a Bio-plex 200 instrument (Bio-Rad Laboratories). Bioplex Manager software version 6 was used to analyze the cytokine concentrations, which are presented as pg/mL/g of tissue. The cytokines values were normalized to tissue weight by dividing observed cytokine concentration (pg/mL) with tissue weight in grams.

### 2.9. Statistical Analysis

Statistical analysis was performed using GraphPad Prism Software version 8.0, California, United States of America). All the data were presented as average mean value ± standard error of mean (SEM) of independent experiments carried out in triplicate or quadruplicate. One-way analysis of variance (ANOVA) followed by Tukey’s post-test was used to evaluate the statistical difference between the three groups. For analysis of body weight change and DAI, Two-way ANOVA followed by Bonferroni’s post-test was used. Data were considered significant when *p* < 0.0001 (****), *p* < 0.001 (***), *p* < 0.01 (**) and *p* < 0.05 (*).

## 3. Results

### 3.1. Idebenone Improves Clinical Symptoms in the Spontaneous Winnie Model of Chronic Colitis

**Figure 1:**
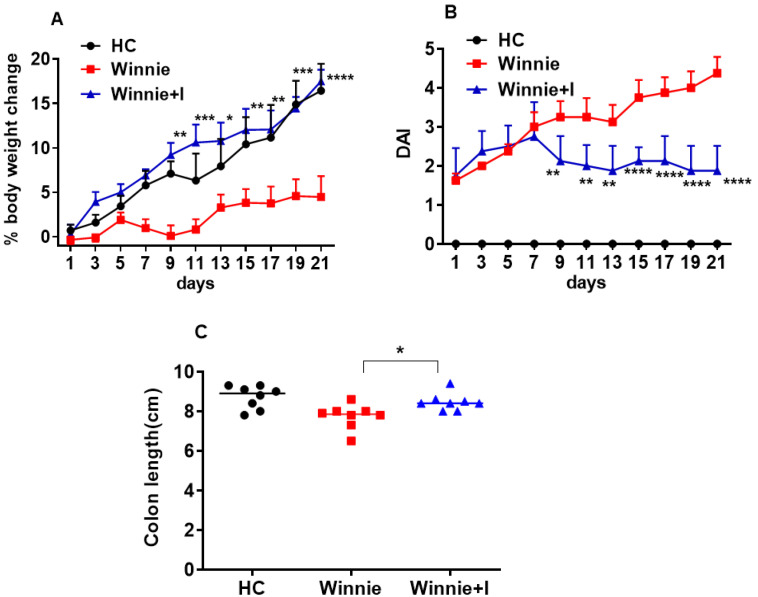
Effect of idebenone on the clinical symptoms of spontaneous chronic colitis. (**A**) % body weight change, (**B**) Disease Activity Index (DAI) and (**C**) Colon length. Data expressed as mean ± SEM (*n* = 8/group). Statistical significance among groups evaluated by Two-way ANOVA followed by Bonferroni’s post-test. * *p* < 0.05, ** *p* < 0.01 *** *p* < 0.001 and **** *p* < 0.0001 vs *Winnie.* Colon length evaluated by One-way ANOVA followed by Tukey’s post-test.

The therapeutic potential of idebenone in spontaneous chronic colitis was determined by measuring the change in body weight, disease activity index (DAI) (integral sum of body weight, bloody stool and stool consistency) and colon length. *Winnie* mice administered idebenone significantly started gaining body weight as early as day 9 (*p* < 0.01). In comparison to *Winnie* animals, which gained 4.5 ± 2.3% body weight, idebenone treatment drastically increased body weight by 17.5 ± 1.3% over the observation period of 21 days, equivalent to *C57BL/6J* healthy controls (HC) (Figure 1A). As compared to HC, DAI increased with age in *Winnie* animals (day 9: 3.3 ± 0.4 and day 21: 4.4 ± 0.4) (Figure 1B). In idebenone-treated *Winnie*, no significant increase of DAI was observed, and from day 9 (2.0 ± 0.2) onwards, DAI was indistinguishable from the start of the treatment.

Macroscopic assessment of the colon clearly indicated the severity of inflammation in *Winnie*; colon was visibly inflamed, thickened and shortened on day 21. In contrast, three weeks of oral administration of idebenone significantly prevented the shortening of the colon (8.5 ± 0.2 cm) when compared to *Winnie* animals (7.7 ± 0.2 cm) (*p* < 0.05) (Figure 1C).

### 3.2. Idebenone Reduces Histopathology in Chronic Colitis

**Figure 2:**
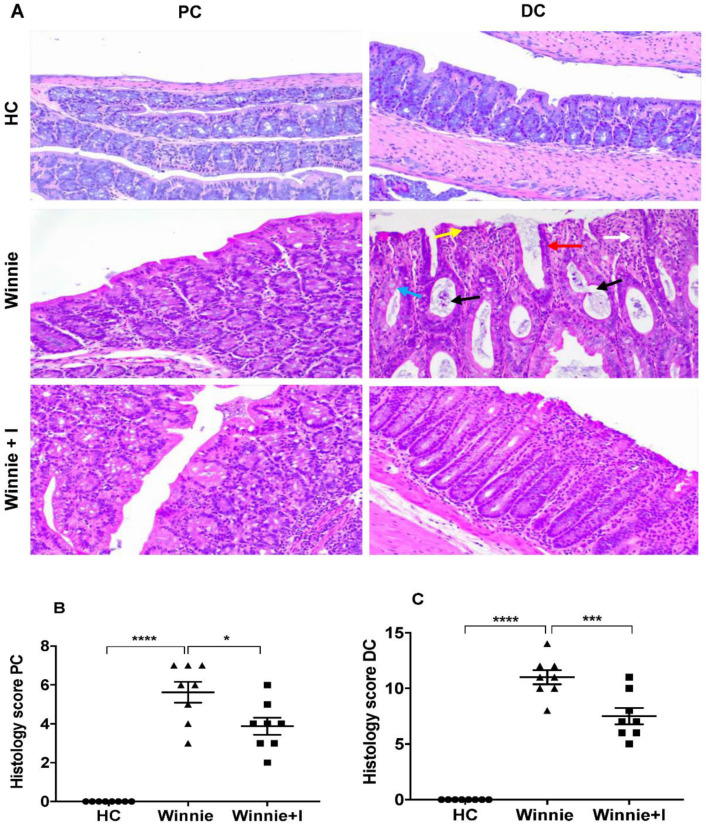
Effect of idebenone on histopathology in the *Winnie* model of spontaneous chronic colitis. (**A**) Representative histological images of proximal (PC) and distal colon (DC) stained with hematoxylin and eosin (H&E) from healthy control *C57BL/J6* mice (HC), untreated *Winnie* mice (Winnie) and *Winnie* mice treated with idebenone (Winnie + I) at 20× magnification. (**B**) and (**C**) Comparison of histology scores for each group in PC and DC. Data expressed as mean ± SEM (*n* = 8/group). Statistical significance among groups was evaluated by One-way ANOVA followed by Tukey’s post-test. * *p* < 0.05, *** *p* < 0.001 and **** *p* < 0.0001. Arrows indicate goblet cells loss (blue), crypt abscesses (black), crypts loss/crypts distortion (red), inflammatory cell infiltration (white) and epithelial surface erosion (yellow).

Spontaneous inflammation of the colon in the *Winnie* model is characterized by severe epithelial surface erosion, distortion of crypt architecture/crypt loss, crypt abscesses, loss of goblet cells, submucosal edema and infiltration of inflammatory cells predominantly affecting distal colon (DC) as compared to proximal colon (PC) (Figure 2A), as revealed from histological analysis. Colon tissue from *C57BL/6J* animals was used as healthy control (HC), with no signs of colon damage (Figure 2A). Treatment of *Winnie* mice with idebenone showed significant protection, particularly in the DC (*p* < 0.001), by reducing the average histology score of untreated *Winnie* animals from 10.7 ± 0.9 to 7.0 ± 0.6 (Figure 2C). In the PC (Figure 2B), the average histology score (5.5 ± 0.6) was also significantly (*p* < 0.05) reduced by idebenone (5.0 ± 0.8). 

### 3.3. Idebenone Restores Mucin (MUC2) Expression in Goblet Cells

**Figure 3:**
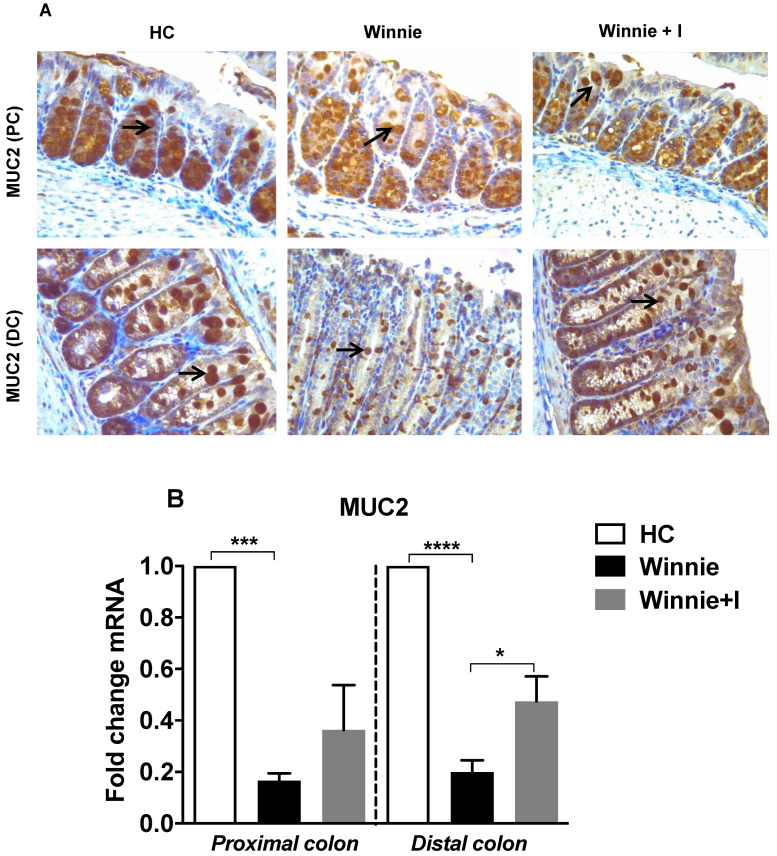
Effect of idebenone on mucin MUC2 expression. (**A**) Representative immunohistochemistry images of MUC2 protein expression in the proximal (PC) and distal colon (DC) (*n* = 3/group) (**B**) *MUC2* mRNA expression (*n* = 4/group). *MUC2* mRNA levels were normalised to GAPDH mRNA and presented as fold change. Statistical significance among groups was determined by One-way ANOVA followed by Tukey’s post-test where * *p* < 0.05, *** *p* < 0.001 and **** *p* < 0.0001. Images were acquired at 40× magnification. Arrows indicate localisation of staining.

Spontaneous chronic colitis in *Winnie* mice is the consequence of a missense mutation in the mucin *MUC2* gene [18]. Immunohistochemical analysis of MUC2 protein expression in the colon of *Winnie* mice confirmed significantly decreased MUC2 expression in both PC and DC goblet cells as compared to colon samples from HC animals (Figure 3A). In the PC, goblet cells containing MUC2 were stained homogenously at the basal as well as the apical surface of crypts, whereas in the DC, goblet cells expressed MUC2 mainly in the apical region. Idebenone treatment in *Winnie* significantly increased MUC2 expression in the DC, whereas only a trend was observed in the PC (Figure 3A). To confirm these results, we examined the *MUC2* mRNA expression (Figure 3B). *MUC2* mRNA was significantly decreased in both the PC and DC of untreated *Winnie* animals when compared to HC animals. In contrast, idebenone treatment upregulated *MUC2* mRNA expression by 2.4-fold in the DC. Although *MUC2* mRNA expression in the PC increased by 2.2-fold compared to the untreated mice, this effect did not reach statistical significance.

### 3.4. Idebenone Reduces Expression of ER Stress Markers

**Figure 4:**
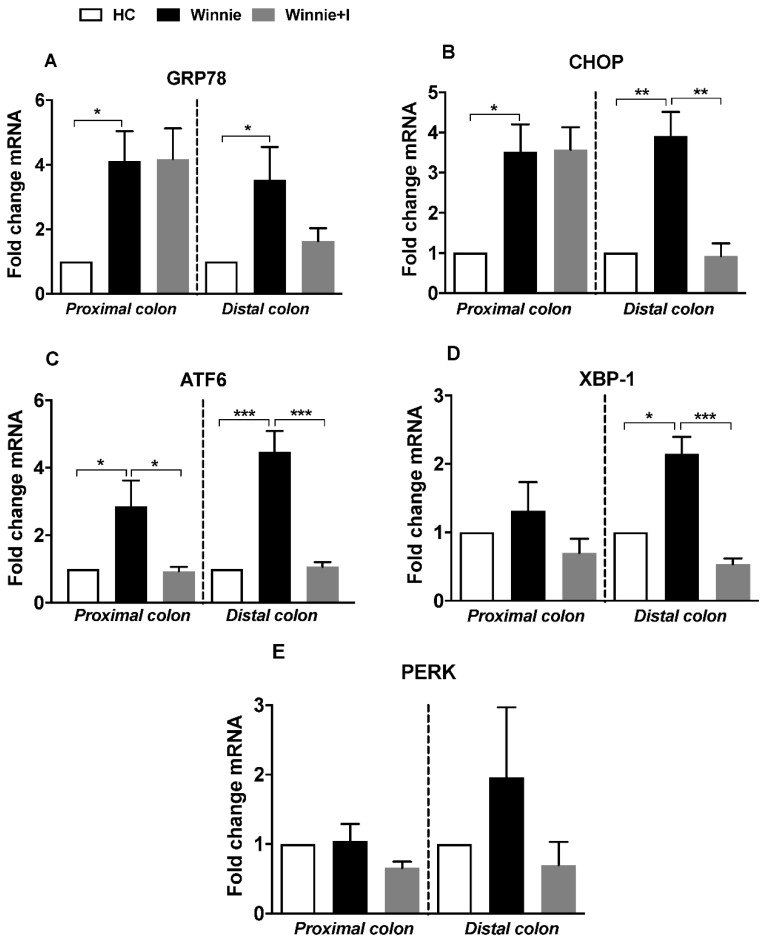
Effect of idebenone on mRNA levels of endoplasmic reticulum stress (ER stress) markers in the *Winnie* model of spontaneous chronic colitis. Gene expression of (**A**) *GRP78*, (**B**) *CHOP*, (**C**) *ATF6*, (**D**) *XBP-1* and (**E**) *PERK* were determined for the proximal (PC) and distal colon (DC) for all the three groups. mRNA levels were normalized to *GAPDH* gene and presented as fold change. Data represents mean ± SEM (*n* = 4/group) using One-way ANOVA followed by Tukey’s post-test, where * *p* < 0.05, ** *p* < 0.01 and *** *p* < 0.001.

Misfolding of MUC2 protein due to reduced biosynthesis and secretion triggers aberrant ER stress, activation of the unfolded protein response (UPR) and spontaneous chronic intestinal inflammation in *Winnie* mice [18]. To explore the influence of idebenone on ER stress, gene expression of ER stress markers was determined by qPCR. Relative to healthy control animals, untreated *Winnie* animals showed significantly increased mRNA levels of key regulators of ER stress, such as chaperon protein *GRP78* (PC: 4.1-fold; DC: 3.5-fold), UPR transcription factor *CHOP* (PC: 3.5-fold; DC: 3.9-fold), UPR signaling molecule *ATF6* (PC: 2.9-fold; DC: 4.5-fold), transcription factor *XBP-1* (PC: 1.3-fold; DC: 2.1-fold) and UPR signaling molecule *PERK* (PC: 1.0-fold; DC: 2.0-fold) (Figure 4A–E).

In contrast, idebenone-treated *Winnie* mice displayed in the DC a significant reduction of *CHOP*, *ATF6* and *XBP-1* mRNAs by 4.3-fold (*p* < 0.01), 4.1-fold (*p* < 0.001) and 4.2-fold (*p* < 0.001) respectively, comparable to the levels of healthy control animals. In the PC, a substantial reduction of *ATF6* mRNA (3.2-fold; *p* < 0.05) was detected, while no significant reductions of *GRP78* and *PERK* mRNAs were detected in either PC or DC compared to untreated *Winnie* mice.

### 3.5. Idebenone Exerts Anti-ER Stress but Not Antioxidant Activity in the Winnie Model

**Figure 5:**
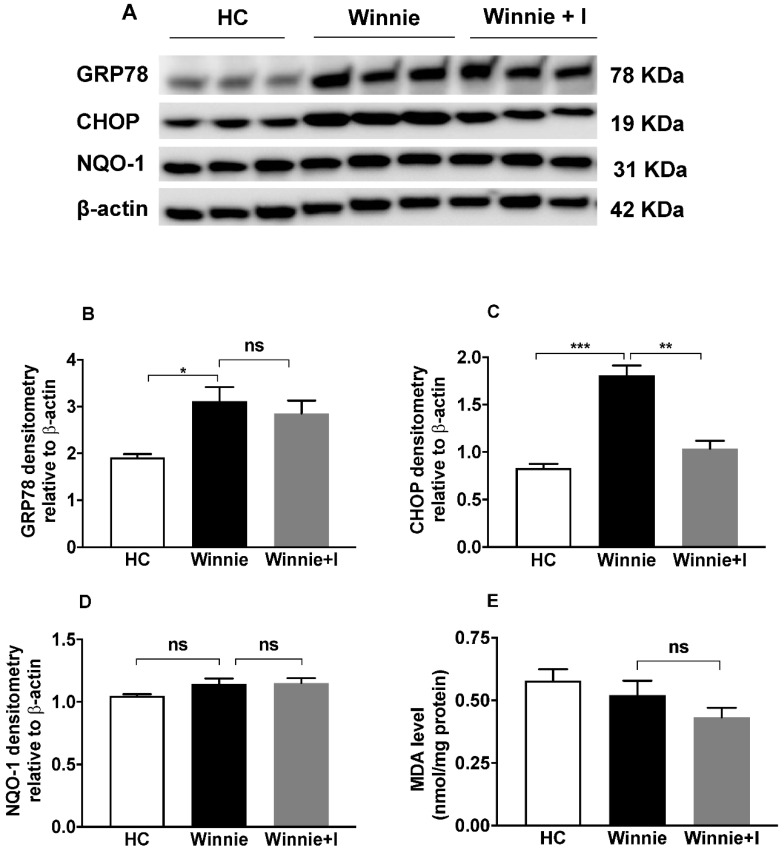
Effect of idebenone on endoplasmic reticulum stress (ER stress) and oxidative stress in spontaneous chronic colitis in *Winnie* mice. (**A**) protein levels of GRP78, CHOP and NQO-1 were analyzed using western blotting of tissue samples from the distal colon (DC), (**B**–**D**) densitometry of GRP78, CHOP and NQO-1 expression. Band densities were normalized to β-actin. (**E**) malondialdehyde (MDA) levels in distal colon. Data represents mean ± SEM (*n* = 3/group). Statistical significance was determined using One-way ANOVA followed by Tukey’s post-test, where * *p* < 0.05, ** *p* < 0.01 and *** *p* < 0.001 and ns: nonsignificant.

Our western blot results of the ER stress markers GRP78 and CHOP also confirmed the reduction of ER stress by idebenone in *Winnie* mice. Idebenone treatment in *Winnie* prevented CHOP upregulation (Figure 5A) but left the expression of GRP78 unaffected (Figure 5A,B). Densitometric analysis of CHOP relative to β-actin also displayed a significant (*p* < 0.01) decrease in CHOP expression (Figure 5C).

To confirm the previously reported antioxidant activity of idebenone [46] in the *Winnie* model, protein expression of the detoxifying enzyme NQO-1 was measured by western blot analysis. Similar levels of NQO-1 were observed in healthy controls, untreated *Winnie* and idebenone-treated *Winnie* animals (Figure 5A), which was confirmed by densitometric analysis (Figure 5D). In addition, due to a lack of elevated levels of MDA (lipid peroxidation marker) in *Winnie* as compared to HC, idebenone treatment in *Winnie* showed no effect on the basal level of lipid peroxidation (Figure 5E).

### 3.6. Idebenone Reduces Pro-Inflammatory Cytokine Levels

**Figure 6:**
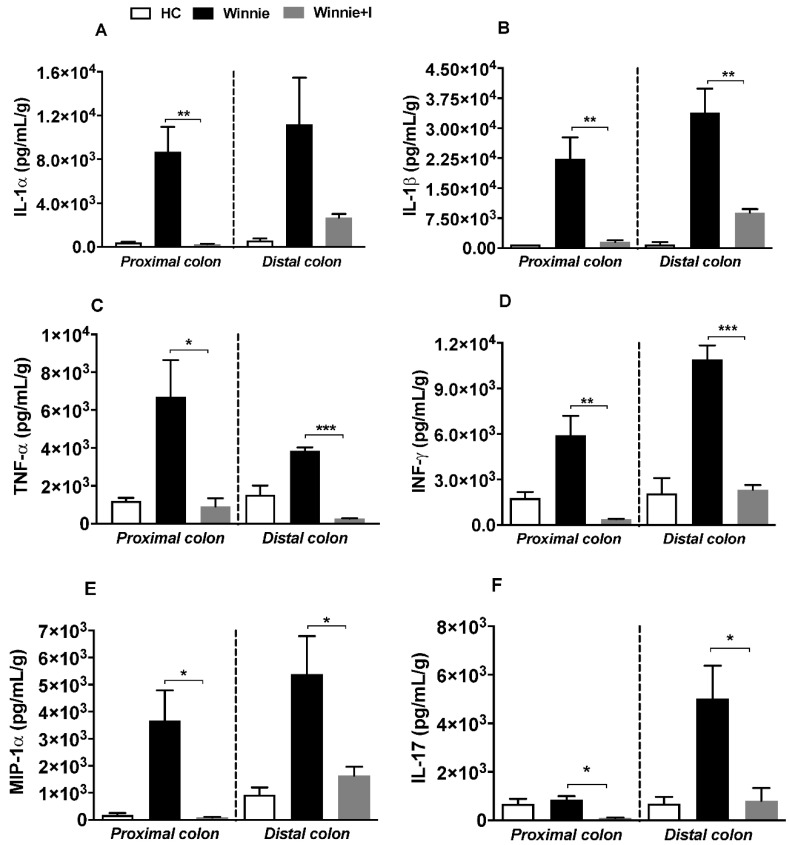
Effect of idebenone on the levels of pro-inflammatory cytokines and chemokines in colonic tissue explants of *Winnie* mice. Tissue levels of (**A**) IL-1α, (**B**) IL-1β, (**C**) TNF-α, (**D**) INF-γ, (**E**) MIP-1α and (**F**) IL-17 in proximal colon and distal colon were quantified by Bio-Plex assay. Data expressed as mean ± SEM (*n* = 3/group) and statistical significance evaluated by One-way ANOVA followed by Tukey’s post-test where * *p* < 0.05, ** *p* < 0.01 and *** *p* < 0.001.

Cytokine analysis was performed to ascertain the anti-inflammatory effect of idebenone in chronic colitis (Figure 6). While colonic explants of untreated *Winnie* animals vigorously secreted pro-inflammatory cytokines (IL-1α, IL-1β, TNF-α, INF-γ, IL-17) and chemokine MIP-1α (macrophage inflammatory protein 1), idebenone treatment considerably suppressed their release in both PC and DC explants of *Winnie* mice. IL-1α was significantly reduced by idebenone-treated PC tissue (*p* < 0.01), while this effect did not reach significance in the DC explants (Figure 6A). A substantial reduction of elevated levels of IL-1β (Figure 6B) was also observed for both idebenone-treated PC and DC tissues at equivalent levels (*p* < 0.01). In addition, idebenone significantly reduced the secretion of TNF-α (*p* < 0.001) and INF-γ (*p* < 0.001) in DC tissues (Figure 6C,D). In the case of idebenone-treated PC sections (Figure 6C,D), the reduction of INF-γ (*p* < 0.01) was more prominent than for TNF-α (*p* < 0.05). Furthermore, idebenone treatment significantly suppressed MIP-1α (*p* < 0.05) (Figure 6E) and IL-17 (Figure 6F) secretion (*p* < 0.05) by both PC and DC explants. No substantial effect was seen for several other cytokines (Supplementary Figure S1).

## 4. Discussion

The current study described the therapeutic potential of idebenone for ameliorating spontaneous chronic intestinal inflammation. The most prominent observation was that idebenone inhibited ER stress, which represents the main causative pathological feature of the *Winnie* model of chronic colitis. Idebenone also improved the histopathology, immune parameters and clinical manifestations of colitis, and increased MUC2 levels.

The current study used idebenone at a dose of 200 mg/kg, which was selected based on a previous study where idebenone ameliorated acute colitis, pro-inflammatory cytokines and oxidative stress in a mouse model of acute colitis [46]. It has to be noted that dose optimization was not the aim of this study, but certainly has to be investigated in future studies to assess the therapeutic potential of idebenone in patients. Idebenone has been reported to be well tolerated by patients, with only minor side effects, and was safely used at doses of up to 2250 mg/day [45]. According to the FDA conversion factor for drug dosing between human and mice, 200 mg/kg in mice would translate to about 1.2 g/day (for 70 kg bodyweight of patient), which is within the clinically used dose range and should therefore not limit the translation of our data to colitis patients [54]. In our preclinical model, idebenone significantly improved body weight, DAI and colon length shortening, similar to results from a DSS-induced model of acute colitis [46]. Similar to the model of acute colitis [46], the histopathology in the present study demonstrated that idebenone protected *Winnie* mice against surface erosion, crypt abscesses, loss of goblet cells, elongation or distortion of crypt architecture and infiltration of immune cells. Surprisingly, at high doses, i.e., >1 g/day, idebenone has been reported to cause gastro-intestinal disturbances such as diarrhea, vomiting and dyspepsia in some patients [55]; these symptoms were not detected in the current study. The reason for this discrepancy is not known at the moment and given that this adverse effect has only been observed in some rare cases, the number of mice used in this study might have been insufficient to detect it.

In the *Winnie* model, spontaneous chronic intestinal inflammation arises as a consequence of a missense mutation in *MUC2* gene, which leads to MUC2 protein misfolding and significantly decreased mucin biosynthesis [18]. Decreased mucus biosynthesis reduces the layer thickness of the mucus barrier, which enables harmful microbes and toxins to penetrate through this intestinal barrier [56]. Consequently, protecting against goblet cell depletion and/or increasing mucus secretion appears to be essential to protect against intestinal inflammation. The present study showed that idebenone increased the mRNA levels of mucin, and also restored mucus production in goblet cells, which confirms a previous study where idebenone also protected mucus expression and prevented loss of goblet cells in a model of acute colitis [46]. In contrast to our study, dexamethasone significantly restored the biosynthesis of mucus in goblet cells by directly reducing UPR and ER stress in *Winnie* mice [24]. Since the present study did not test a direct connection between MUC2 biosynthesis, ER stress and UPR, this restoration of mucus by idebenone could either be a consequence of its anti-inflammatory activity or a direct effect of idebenone on goblet cells to increase MUC2 biosynthesis. A detailed understanding of the molecular mode of action of idebenone should be sought in future studies that should include a detailed analysis of MUC2 biosynthesis, as well as goblet cell differentiation/maturation.

Several preclinical and clinical studies support a central role of ER stress in the pathogenesis of UC [25,57,58]. During ER stress, the heat shock protein GRP78 is upregulated and dissociated from the UPR mediator ATF6, which activates a cascade of molecular events that leads to the transcription of UPR genes. After release from GRP78, ATF6 migrates to the Golgi apparatus, where it is proteolytically activated before it translocates to the nucleus, where it induces the expression of transcription factors CHOP and XBP-1 [26,28,31,33,59,60,61,62,63,64]. Previous reports of increased expression of *GRP78*, *PERK*, *CHOP*, *XBP-1* and *ATF6* in colonic tissues of IBD patients and in *Winnie* mice [18,24,25] support the data presented in this study. However, idebenone treatment did not significantly reduce *GRP78* and *PERK* expression, which makes them unlikely targets for idebenone. Therefore, it appears unlikely that *GRP78* and *PERK* were involved in reducing ER stress in the present study.

The upregulation of ATF6-induced transcription factor CHOP plays a major role in mediating ER stress-induced apoptosis and epithelial cells proliferation [65], which is replicated in the *Winnie* mouse model [18]. Interestingly, idebenone was reported to reduce *CHOP* expression in a mouse model of mitochondrial disease, which was associated with improved motor function [49]. Moreover, a recent study confirmed the reduction of *CHOP* gene expression by idebenone in mutant myocilin cells, which was associated with antiapoptotic effects [66]. The present study supports this effect by demonstrating the downregulation of *CHOP* mRNA and its protein levels. In addition, it adds to previous studies by providing a mechanistic clue that idebenone also downregulates the expression of *ATF6*, which is the upstream signaling molecule of *CHOP*. This effect was confirmed by the fact that idebenone also reduced *XBP-1* expression, which is also controlled by ATF6. A previous study reported that glucocorticoids also reduce the expression of *CHOP*, *XBP-1* and *ATF6* in *Winnie* mice, as well in an in vitro model of tunicamycin-induced ER stress, which parallels our results in the present study [24]. Overall, our results suggest that idebenone alleviates ER stress by targeting the *ATF6/CHOP/XBP-1* axis. However, this study did not clarify whether idebenone targets CHOP directly or indirectly by affecting genes upstream of CHOP; this will have to be addressed in future studies.

Excessive accumulation of ROS disrupts cellular functions by the peroxidation of lipids, a process that can lead to gastrointestinal disturbances [15]. Under physiological conditions, the harmful effects of excess ROS production are counteracted by endogenous antioxidant defense mechanisms [67]. This cellular antioxidant machinery includes phase II detoxifying enzymes such as NQO-1, SOD and glutathione reductase, glutathione peroxidase and hemeoxygenase-1 (HO-1), which are regulated by the transcription factor nuclear factor erythroid 2-related factor 2 (Nrf-2) [15,68]. Previous results in a model of acute colitis associated increased lipid peroxidation (MDA) with decreased levels of NQO-1 and SOD [46]. However, the present study did not observe increased levels of lipoperoxidation in the *Winnie* mice. In line with this result, NQO1 levels of and SOD activity were indistinguishable between healthy control and *Winnie* mice, which indicates that the *Winnie* model in the early stages is not associated with excess levels of ROS. Consequently, idebenone did not affect the basal levels of lipid peroxidation and NQO-1 in *Winnie* mice. Overall, our results therefore indicate that idebenone either utilizes different protective pathways in models of acute versus chronic colitis or that a common, as yet undiscovered mode of action is responsible for initiating different protective responses.

Immune system dysregulation due to abnormal secretion of pro-inflammatory cytokines and chemokines is the hallmark of UC pathogenesis [69]. Intestinal inflammation involves immune cells of both the innate and adaptive immune systems, and is characterized by marked increases in pro-inflammatory cytokines IL-1β, TNF-α, IL-12, IL-17, IL-1α and INF-γ [70]. Specifically, increased levels of cytokine IL-1β and the correlation of this with disease severity have been reported in IBD patients [71]. TNF-α, a pleiotropic cytokine, instigates its inflammatory action by inducing the secretion of additional pro-inflammatory cytokines such as IL-1β in mice as well as in IBD patients [72,73]. Similarly, INF-γ and chemokine MIP-1α have also been observed to be highly overexpressed in the mucosa of UC patients [74,75]. The contribution of pro-inflammatory cytokine IL-17 is well defined in IBD. Elevated levels of IL-17-producing immune cells and excessive production of Th-17 cells-mediated pro-inflammatory cytokines have been reported in UC patients [76,77]. In this study, *Winnie* mice displayed substantially elevated levels of IL-1α, IL-1β, TNF-α, INF-γ, IL-17 and MIP-1α as compared to *C57BL/6J* healthy controls, which is in alignment with previous reports [18,24,37,40,41,46]. Idebenone treatment suppressed elevated levels of these cytokines to their baseline level in *Winnie* colon. This idebenone-induced anti-inflammatory property was also observed in multiple disease models including DSS-induced acute colitis, LPS-mediated neuroinflammation in BV2 microglial cells and in renal damage induced by titanium dioxide in rat, where idebenone suppressed certain pro-inflammatory cytokines and chemokines [46,47,48]. Our results in *Winnie* further support the reports mentioned above regarding the anti-inflammatory action of idebenone.

Growing evidence points to a direct intersection between immune response and ER stress in inflammatory disorders [18,25,78,79], whereby inflammation induces ER stress and vice versa. The ER stress-inflammation connection results in a vicious cycle that propagates chronic inflammation. For example, elevated levels of TNF-α and INF-γ initiated ER stress via increasing the expression of *GRP78* in Caco2 intestinal cell lines [80]. Furthermore, IL-17A signaling induced ER stress by increasing the expression of *CHOP* and *GRP78* in LPS-induced lung injury [81]. On the other hand, UPR mediated ER stress directly activates nuclear factor kappa-light-chain-enhancer of activated B cells (NF-κB) transcription factor and pro-inflammatory cytokines [82,83,84,85,86]. Based on our research, it was hypothesized that idebenone provides protection against chronic intestinal inflammation by simultaneously suppressing ER stress and pro-inflammatory cytokines. However, we still do not know the hierarchical order, i.e., whether idebenone first exerts an immunomodulatory action directly or indirectly through general protection against ER stress. Overall, we believe that idebenone has properties that act against existing ER stress, potentially allowing the goblet cells in the colon to retain their function in addition to mopping up the milieu of cytokines in the mucosa.

**Figure 7:**
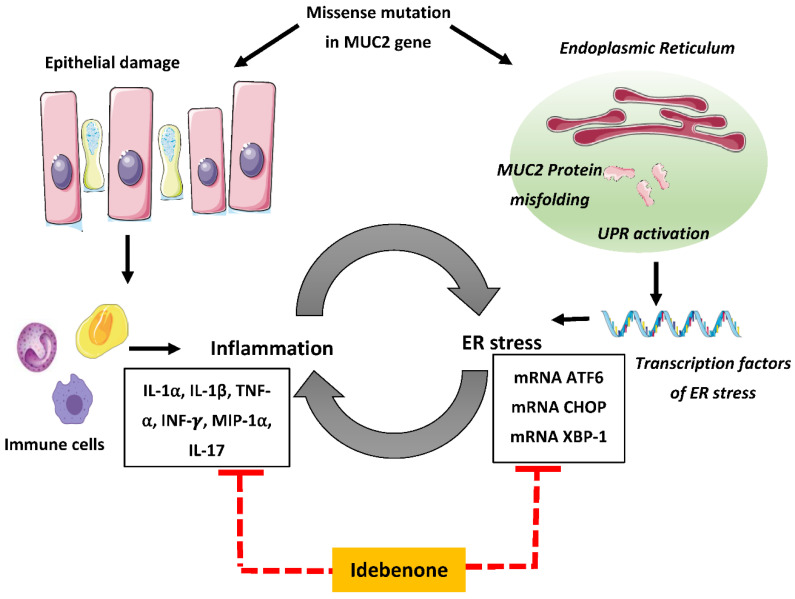
Schematic illustration of proposed mechanism of action of idebenone in chronic colitis. A missense mutation in the mucin *MUC2* gene of secretory epithelial goblet cells damages the epithelial surface, reduces mucus production and provokes spontaneous inflammation. Inflammation exacerbates ER stress and UPR activation, creating a vicious cycle of chronic inflammation. The administration of idebenone for 3 weeks in *Winnie* restores *MUC2* expression, lessens ER stress via suppressing the *ATF6/CHOP/XBP-1* axis and reduces elevated levels of pro-inflammatory cytokines. Idebenone protected against chronic intestinal inflammation in *Winnie* mice by suppressing ER stress and pro-inflammatory cytokines. ER: endoplasmic reticulum; IL: interleukin; TNF-α: tumor necrosis factor alpha; INF-γ: interferon gamma; MIP-1α: macrophage inflammatory protein 1 alpha; UPR: unfolded protein response; ATF6: activating transcription factor 6; CHOP: C/EBP homologous protein; and XBP-1: X-box binding protein 1.

To our knowledge, the present study is the first to highlight the potential of idebenone to ameliorate chronic intestinal inflammation by effectively reducing ER stress. The beneficial effects of idebenone in this model of spontaneous chronic colitis were associated with the suppression of ER stress, the restoration of *MUC2* expression and the simultaneous reduction of increased levels of pro-inflammatory cytokines (Figure 7). It can be speculated that the synergism between these activities was responsible for remarkably reducing the severity of chronic intestinal inflammation in *Winnie* mice. Despite the strong anti-inflammatory effect and mucosal healing that was observed with the administration of idebenone, the major shortcoming of this study is the lack of a molecular target. Studies designed to specifically address each component (ER stress, mitochondrial stress and cytokines) will be required to identify the specific pathways targeted by oral administration of idebenone. The results of the present study establish the possible importance of idebenone therapy, i.e., in targeting multiple factors linked to UC pathology including epithelial ER stress, mucin production and inflammatory markers. Therefore, idebenone could be considered a promising therapeutic alternative to treat the chronic phases of multifaceted UC.

## Supplementary Materials

Supplementary materials can be found at https://www.mdpi.com/2227-9059/8/10/384/s1, Figure S1: Effect of idebenone on the levels of pro-inflammatory cytokines and chemokines in colonic tissue explants of Winnie mice.
