# Supplementary material for: Idebenone Protects against Spontaneous Chronic Murine Colitis by Alleviating Endoplasmic Reticulum Stress and Inflammatory Response
Source: Biomedicines. 2020 Sep 28;8(10):384. doi: 10.3390/biomedicines8100384 (PMC7601570; doi:10.3390/biomedicines8100384)
Supplement: Supplementary file 1 [file biomedicines-08-00384-s001.pdf]

Idebenone protects against spontaneous chronic murine colitis by alleviating endoplasmic reticulum stress and inflammatory response

Authors: Sonia Shastri[1*], Tanvi Shinde[2], Agampodi Promoda Perera[1], Nuri Gueven[3], Rajaraman Eri[1*]

[1]Gut Health Laboratory, School of Health Sciences, College of Health and Medicine, University of Tasmania, Launceston, Tasmania 7250 Australia

[2]Centre for Food Safety and Innovation, Tasmanian Institute of Agriculture, University of Tasmania, Launceston, Tasmania 7250 Australia

[3] School of Pharmacy and Pharmacology, College of Health and Medicine, University of Tasmania, Hobart, Tasmania 7005 Australia

***Correspondence:**

Sonia Shastri
Sonia.Shastri@utas.edu.au (Mobile: +61-449924236)

Rajaraman Eri
Rajaraman.Eri@utas.edu.au (Tel: +61-3-6226-5017)

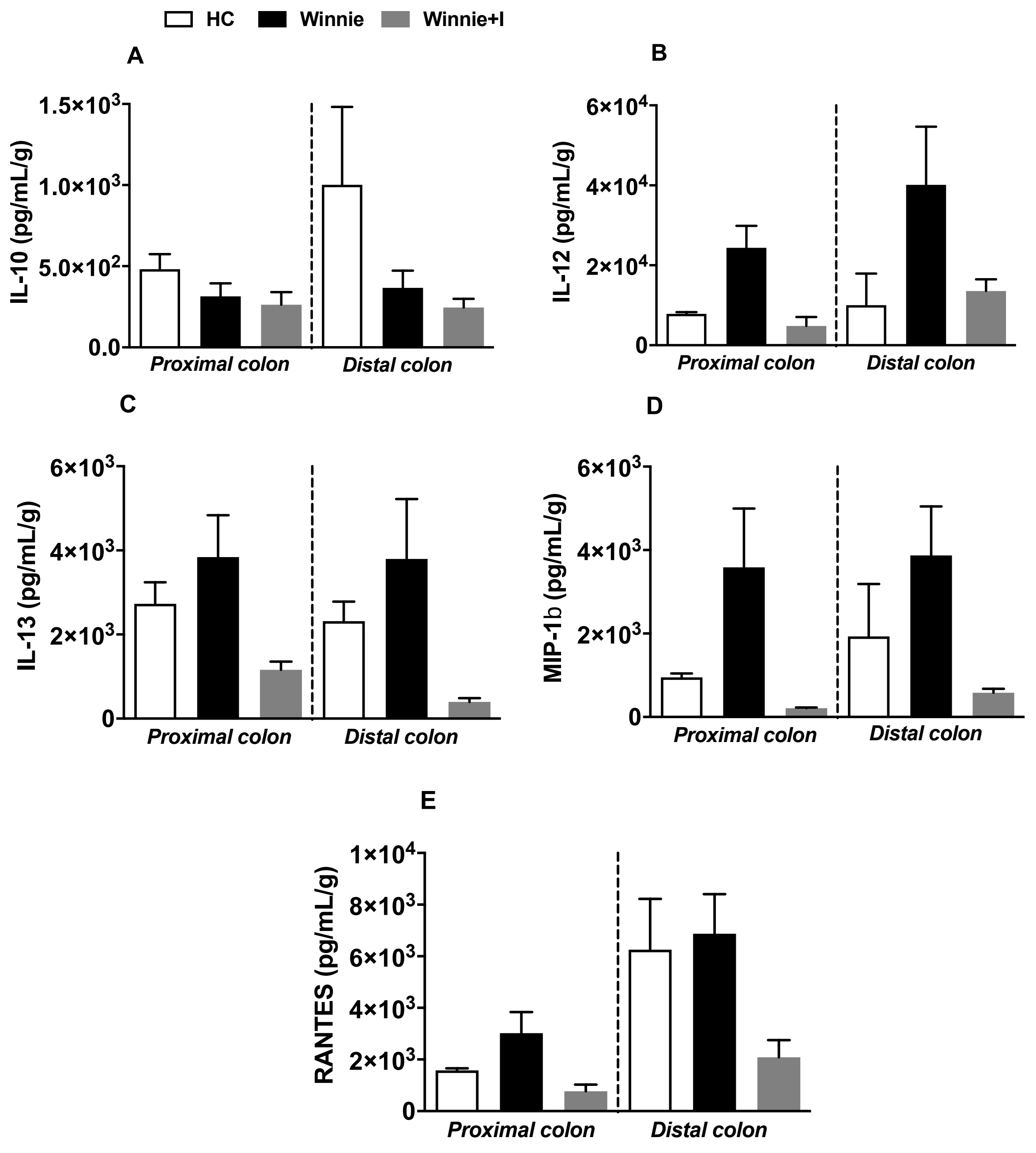

Figure S1. Effect of idebenone on the levels of pro-inflammatory cytokines and chemokines in colonic tissue explants of Winnie mice. Tissue levels of A) IL-10, B) IL-12, C) IL-13, D) MIP-1β and E) RANTES in proximal colon and distal colon were quantified by Bio-Plex assay. Data expressed as mean ± SEM (n=3/group) and statistical significance evaluated by One-way ANOVA followed by Tukey's post-test.
